# The association between healthcare expenditures and potentially inappropriate medication use in hospitalized older adults in Ethiopia

**DOI:** 10.1186/s12877-024-04688-w

**Published:** 2024-01-16

**Authors:** Behailu Terefe Tesfaye, Dula Dessalegn Bosho, Gashahun Mekonnen Dissassa, Mikiyas Gashaw Tesfaye, Mengist Awoke Yizengaw

**Affiliations:** 1 Department of Clinical Pharmacy, School of Pharmacy, Faculty of Health Sciences, Institute of Health, Jimma University, Jimma, Ethiopia; 2 Department of Internal Medicine, School of Medicine, Institute of Health, Jimma University, Jimma, Ethiopia; 3 Department of Laboratory, Clinical Chemistry Unit, Jimma Medical Center, Jimma, Ethiopia

**Keywords:** Aged, Potentially inappropriate medication, Healthcare costs, Ethiopia

## Abstract

**Background:**

Evidence shows that potentially inappropriate medication (PIM) use in older adults significantly increases the utilization of healthcare resources. PIM is widely prescribed in older adults, however little is known about its association with healthcare resource utilization in Africa, particularly Ethiopia. Therefore, the primary aim of this study was to examine the presence of an association between healthcare expenditure and the frequency of PIM used.

**Methods:**

This observational study was conducted from 06 September 2021 to 30 December 2022. A total of 151 hospitalized older adult patients were included in the study. The data collection format was designed to capture relevant information. STATA V.15.0 was used for analysis. Descriptive statistics and a generalized linear model regression were conducted. Statistical significance was set at a *p*-value < 0.05. The findings are presented in tables, figures, and text.

**Results:**

The total healthcare expenditure was higher in PIM users (385,368.6 ETB) than in non-PIM users (131,267.7 ETB). The median expenditures for medical supplies (*p* = 0.025), investigations (*p* = 0.033), and total healthcare expenditure (*p* = 0.023) were significantly higher in patients with two and more PIMs than in those with no or one PIM. However, after model adjustment, the total healthcare expenditure was not significantly different across the frequency of PIMs used. Number of medications (adjusted B = 0.068, 95% CI: 0.035, 0.101, *p* < 0.001) and taking blood and blood-forming organ class of medication (adjusted B = 0.151, 95% CI: 0.005, 0.297, *p* = 0.042) were associated with higher total healthcare expenditure, whereas the total healthcare expenditure was significantly lower in those patients with diseases of the nervous system (adjusted B = -0.307, 95% CI: -0.502, -0.112, *p* = 0.002) and respiratory system (adjusted B = -0.196, 95% CI: -0.327, -0.065, *p* = 0.003).

**Conclusion:**

The total healthcare expenditure is nearly three times higher in PIM users. However, its association with the frequency of PIM use was not statistically significant in the final model. Deprescribing medications after evaluating the benefit-risk ratio may help to reduce the healthcare expenditures of older adult patients. Further similar, adequately powered, comparative study is also warranted to identify the actual effect of PIM use on healthcare expenditures in the local context.

## Background

The proportion of the older adult population across the globe is increasing over time [1]. It is projected that in 2050, approximately 80% of the older adult population will be living in low- and middle-income countries [2]. As the majority of healthcare resources are spent on this age group [3], a further enormous impact is expected on the healthcare system in line with the rapidly evolving demographic change [4]. This impact is more pronounced in developing countries, such as Ethiopia, where healthcare resources are scarce [5, 6].

The increased risk of multimorbidity, polypharmacy, and vulnerability to drug-related problems [7–9] are among the factors that increase the consumption of healthcare resources in older adult care. Furthermore, reports revealed an increased risk of healthcare resource utilization with the use of potentially inappropriate medications (PIMs) [10–12]. Multiple studies from developed countries reported higher healthcare expenditures in older adult patients who received PIM than in patients who did not [13–17]. For instance, a retrospective study from the United States of America revealed that the total cost of patients receiving PIMs was more than twice that of nonusers ($4,472.43 versus $2,065.38) [13]. In another retrospective study from Germany, the mean annual total healthcare costs per individual in the PIM-exposed group exceeded those in the nonexposed group by €2321 [10]. In Japan, a retrospective study recorded a 33% higher medical cost in PIM users than in nonusers [16]. A longitudinal study from Canada also showed higher healthcare costs ($3,076 higher per year) in patients with PIM [15]. In Finland, a longitudinal study recorded a 15% higher hospital cost in PIM users compared to nonusers (38). These findings are also corroborated by a systematic review in which PIM use was reported to increase the cost per older adult patient by $2000 [18].

In Africa, the rate of PIM use in older adult patients is as high as 68.9% [19–26]. Although there are multiple pieces of evidence on the adverse consequences of PIM use on healthcare resource utilization in other parts of the globe, to date, little is known in Africa, particularly in Ethiopia. Due to the possibility of variation in medication availability as well as the healthcare system, the projection of findings of the previous studies from other parts of the world to Ethiopia would be unreliable. This urges for similar local evidence. Based on the previously available study findings in other parts of the globe [10–18, 27], we hypothesized that there will be an association between PIM use in hospitalized older adults and their direct healthcare expenditure.

## Methods

### The aim, design and setting of the study

This prospective observational study was conducted with the primary intention of generating local evidence on the association between the frequency of PIM use and healthcare expenditure in hospitalized older adult patients in Ethiopia. Describing the prevalence of PIM, specific types of PIMs prescribed, specific categories of healthcare expenditure (bed day cost, medication cost, medical supply cost, and investigation cost), and and other factors associated with the total healthcare expenditure were the secondary objectives of the study. Data were collected from September 6, 2021, to December 30, 2022. The study was conducted in the medical wards of Jimma University Medical Centre (JUMC). The JUMC was established in 1930 in Jimma town, 352 km southwest of Addis Ababa. It is the only teaching and referral hospital in the southwestern part of Ethiopia, with a bed capacity of 800. It provides services for approximately 16,000 inpatient and 220,000 outpatient clients a year with a catchment population of approximately 15 million people [28].

### Study participants’ characteristics

All older adult patients admitted to the medical wards of JUMC were considered as the source population, and the study participants were consented older adult patients (60 years and above) admitted to the medical wards (general unit, cardiac unit, stroke unit, and pulmonary unit).

### Data collection tool and procedure

After an in-depth review of the relevant literature, the data collection tool was designed to capture the sociodemographic, behavioural, functional, clinical, medication, healthcare expenditures, and related information of the participants. The tool was translated into two locally predominant languages (Afan Oromo and Amharic) and back-translated into English to check the consistency. The data collectors (two pharmacists with master's degrees in clinical pharmacy and one nurse with a bachelor's degree) were trained on the data collection tool and procedure. A pretest was conducted before the actual data collection. The investigators closely supervised the data collection procedure throughout the study period. All eligible patients were enrolled at admission to the ward and followed strictly during the hospital stay. Patient charts, laboratory results, patient/caregiver interviews, and practitioners in charge were the sources of data for the present study. Body mass index (BMI) was calculated using the standard formula, BMI = weight in kg/ (height in m) ^2^. The weight of the study participants was measured using a beam balance. For taking the weight of the participant, each of them was requested to take off their shoes and heavy clothes, including jackets, jerseys, and belts; the balance was calibrated, and the figure was approximated to the nearest 0.01 kg. For measuring height, a seca vertical height scale was used. Patients were asked to take off their shoes and stand upright in the middle of the board. Thereafter, height was measured to the nearest 0.01 cm by making the participant`s back of the head, the shoulder blades, the buttocks, and the heels touch the measuring board. Upon encountering patients who cannot erect upright because of their illness, their height was estimated from demi-span. In line with a standard recommendation [29], the length from the sternal notch to the finger roots (demi-span) was measured by a flexible validated plastic tape with the patient sitting upright or recumbent depending on the patient’s abilities. Height was estimated as follows: for females: Height in cm = (1.35 × demi-span in cm) + 60–1, for males: Height in cm = (1- 40 × demi-span in cm) + 57.8.

### Independent variables

The independent variables were sociodemographic and related variables (age, sex, residence, marital status, educational level, patient’s current working status, current occupation, and financial dependence), behavioural, functional, and related information (alcohol consumption history, cigarette smoking history, khat chewing history, recent traditional medicine use history, cohabitation, activities of daily living, body mass index, kg/m2), and clinical and related information (past medical history, hospitalization in the previous year, psychological condition at admission, current diagnoses, number of diseases diagnosed, Charlson comorbidity index (CCI), length of hospital stay, and medication and related information (past medication history, in-hospital medications, polypharmacy (≥ 5 medications per patient)). The Katz Index of Independence in Activity of Daily Living (ADL) was employed to assess functional health status (disability) at admission [30]. The tool ranks adequacy of performance in six functions (eating, dressing, bathing, transferring, continence and toileting), with each rank assigned a score of 1 or 0. The overall patient ranking is a Katz score of 6 = independent (full function), 3–5 = partially dependent (moderate impairment), and 2 or fewer points = dependent (severe functional impairment) [30, 31]. Psychological condition at admission was objectively assessed using the shortened self-report form of the Geriatric Depression Scale (GDS), which comprises 15 items [32]. Patients respond in a "Yes/No" format. The patients are categorized as having no psychological problem (0 to 4), mild depression/dementia (5 to 9) or severe depression/dementia (10 to 15). Diseases were categorized according to the International Classification of Diseases (ICD)-11 system [33], while MDCalc online was employed to calculate the CCI score [34]. Patients were categorized into mild, with CCI scores of 1–2; moderate, with CCI scores of 3–4; and severe, with CCI scores of ≥ 5 [35]. The Anatomical Therapeutic Chemical (ATC) system is employed to categorize medications [36].

### Outcome variable

The primary objective of the study is to assess the presence of an association between PIM frequency and total healthcare expenditure. Total healthcare expenditure was operationalized as the sum of medication/prescription cost, bed cost, investigation cost, and cost of medical supplies. Prevalence of PIM, specific types of PIMs prescribed, categories of healthcare expenditure (bed day cost, medication cost, medical supply cost, and investigation cost), and other factors associated with the total healthcare expenditure are the secondary outcomes.

### Outcome measurements

From a patient perspective, the cost valuation was carried out by a direct allocation method. The study considered healthcare expenditure costs patients spent to gain service and/or items at the inpatient level. Bed days expenditure was calculated by multiplying the bed days of the patient by bed cost/day. Medication (prescription) cost, medical supply cost, and investigation cost were each determined by summing up all costs the patient spent buying medications and medical supplies and undergoing investigations during the whole stay in the medical wards. In the present study, the term investigation expenditure refers to the payment made by the patient to undergo imaging studies (such as X-ray, CT scan, magnetic resonance imaging, ultrasound, and endoscopy) and laboratory tests. For patients who have health insurance coverage, all corresponding healthcare expenditures were calculated by allocating the price of each item (medication, medical supply) and services (bed, laboratory and imaging investigations). For such individuals, the price for services and items was allocated per the centre`s price lists obtained from the pharmacy unit, laboratory/investigation unit, and finance office. Finally, the total healthcare expenditure was determined by summing the bed day cost, medication cost, medical supply cost, and investigation cost in Ethiopian birr (ETB) over the hospital stay. The cost program medications, such as anti-tuberculosis and anti-retroviral medications, were not included in the medication cost estimation. According to the National Bank of Ethiopia`s report, the commercial bank`s weighted average exchange rate of one US dollar to ETB at the start of this study (06 September 2021) was $1 = 45.6011 ETB, while it was $1 = 53.2566 ETB at the end of the study period (30 December 2022). On 01 March 2023, the exchange rate was $1 = 53.7535 ETB [37]. The 2019 updated AGS Beers Criteria® [38] was employed to assess PIM use. This criteria is used as a guide to improve medication selection, educate clinicians and patients, reduce adverse drug events, and evaluate quality of care, cost, and patterns of drug use among older adults. The Beers tool consists of the following criteria: medications that are potentially inappropriate in older adults, those that should typically be avoided in older adults with certain conditions, drugs to use with caution, drug-drug interactions, and drug dose adjustment based on kidney function. Though PIM can also be assessed using tools other than Beers, in the present study, Beers tool was used to incorporate a more extensive list of PIMs as compared to others. The AGS Beers Criteria are also widely used by healthcare providers, researchers, and educators. This tool has been used in most previous studies from Ethiopia [21, 23, 24, 39, 40]. It should be noted that, while using the AGS Beers Criteria®, clinicians should consider patient-specific factors when determining if a medication is to be discontinued, modified, or added.

### Sample size and sampling technique

From the patient registration book review, the number of older adult patients aged 60 years and above admitted to the medical wards of JUMC in a year (2019–2020) just before the study period was 398. Since there were no locally available previous studies on the association between healthcare expenditure and PIM frequency, with the assumption of ensuring adequate power of the study, we planned to include all eligible older adults who will be admitted over the period of one year starting from September 06, 2021. However, during the actual study period (a period during which COVID-19 was a challenge to the healthcare system), there were very limited admissions of older adults to the medical wards of JUMC. Therefore, we could not achieve the number of participants we planned even after extending the study for four months (one year and four months). A total of 176 hospitalized older adult patients were consecutively approached over the study period and 25 were rationally excluded from the study (2 patients with aphasia problem, 12 readmissions, and 11 patients lost to follow-up). Finally, 151 consented older adult patients admitted to the medical wards of JUMC in the study period were considered for analysis.

### Data management and statistical analysis

Data were checked for completeness and accuracy regularly during the data collection period, entry, and before the analysis. Epi data version 4.2.0.0 and STATA V.15.0 were employed for data entry and analysis, respectively. Categorical variables were described using frequency and percentage. Performing the Shapiro–Wilk test, the distribution of continuous variables does not follow a normal (Gaussian) distribution, thus described in median and interquartile (IQ). The Kruskal‒Wallis test was conducted to evaluate the distribution of healthcare expenditures across the frequency of PIMs taken by the patients over the hospital stay. The healthcare expenditure was right skewed in distribution toward larger values. For such kinds of variables with positive scale values with positively-skewed data, it is reasonable to use a generalized linear model (GLM) with a log link and gamma variance. Hence, a GLM with a log link and gamma variance was conducted to determine the association between healthcare expenditure and the number of PIMs. Covariates with a *p*-value < 0.25 in bivariable regression were used to adjust the association between healthcare expenditure and PIMs in multivariate regression. Multicollinearity was checked using the variance inflation factor (VIF), and all factors included in the multivariate regression had a VIF of < 10 (maximum 2.16). In all the analyses, a *p*-value of < 0.05 was used to declare statistical significance.

## Results

The median age of the participants was 65 years; 123 (81.5%) were males and 49 (32.5%) had an occupation. Though the majority of the participants had no occupation, 117 (77.5%) participants declared financial independence for healthcare expenditures. Almost all participants, 144 (95.4%), reported to live with families. Physical independence assessment indicated that over two-thirds, 107 (70.9%), of the participants require assistance to carry out their ADL. In terms of BMI, nearly two-thirds of the participants, 99 (65.6%), were in a healthy weight range (Table 1).

**Table 1:** Sociodemographic and related information of the study participants

Sociodemographic and behavioural variables		Frequency (%)
Age (in years), Median (IQ)		65 (60, 65)
Sex	Male	123 (81.5)
	Female	28 (18.5)
Residence	Urban	31 (20.5)
	Rural	120 (79.5)
Marital status	Married	125 (82.8)
	Divorced	8 (5.3)
	Widowed	18 (11.9)
Educational level	Cannot read and write	111 (73.5)
	Nonformal education	32 (21.2)
	Primary education (1–8 grade) and above	8 (5.3)
Patients currently working	Yes	49 (32.5)
	No	102 (67.5)
Current occupation	Retired	20 (13.3)
	Employed	1 (0.7)
	Housewife	23 (15.2)
	Private work	48 (31.8)
	Nonemployed	59 (39.1)
Financial dependence	Dependent	34 (22.5)
	Independent	117 (77.5)
Alcohol drinking	Never	109 (72.2)
	Previously	42 (27.8)
Cigarette smoking	Never	111 (73.5)
	Ex-smoker	38 (25.2)
	Current	2 (1.3)
Khat chewing	Never	44 (29.1)
	Previously	96 (63.6)
	Current	11 (7.3)
Traditional medicine use history		21 (13.9)
Cohabitation	Live with family	144 (95.4)
	Live alone	7 (4.6)
Activities of daily living	Dependent	59 (39.1)
	Partially dependent	48 (31.8)
	Fully independent	44 (29.1)
Body mass index, kg/m^2^	Underweight (< 18.5)	44 (29.1)
	Normal (18.5 to < 25)	99 (65.6)
	Overweight (25.0 to < 30)	8 (5.3)

The participants` clinical information shows, 97 (64.2%) had at least one medical problem, and 50 (33.2%) had a hospitalization history in the previous year. Upon assessing psychological condition on admission based on the GDS score, over three-fourths, 118 (78.1%), of the participants had psychological problems. Overall, mental and behavioural disorders, 118 (78.2%), were the most frequent diagnoses, followed by diseases of the circulatory system, 105 (69.5%). Over the hospital stay, about one-third of participants, 38 (28.2%), were on polypharmacy (≥ 5 medications). The most frequently prescribed medications were from the cardiovascular system class, 113 (74.8%), followed by anti-infectives for systemic use, 104 (68.9%). Overall, the participants stayed inpatient for a median duration of 10 days (Table 2).

**Table 2:** Clinical and related information of the study participants

Clinical and related information		Frequency (%)
Patients with a previous medical history		97 (64.2)
Hospitalization in the previous one-year		
None		101 (66.9)
Ones		46 (30.5)
Twice		4 (2.7)
Psychological condition on admission		
Severe dementia or depression		28 (18.5)
Mild dementia		90 (59.6)
No psychological problems		33 (21.9)
Currently diagnosed diseases according to ICD-11 classification		
Certain infectious or parasitic diseases		19 (12.6)
Neoplasms		3 (2.0)
Diseases of the immune system		4 (2.7)
Endocrine, nutritional or metabolic diseases		35 (23.2)
Mental, behavioural or neurodevelopmental disorders		118 (78.2)
Diseases of the nervous system		20 (13.25)
Diseases of the circulatory system		105 (69.5)
Diseases of the respiratory system		68 (45.0)
Diseases of the digestive system		12 (8.0)
Diseases of the skin		1 (0.7)
Diseases of the blood or blood-forming organs		30 (19.9)
Diseases of the genitourinary system		34 (22.5)
Symptoms, signs, or clinical findings, not elsewhere classified		12 (8.0)
Number of diseases diagnosed, Median (IQ)		3 (3, 3)
Minimum, Maximum per patient		1, 8
Charlson comorbidity index, Median (IQ) score		4 (3, 5)
Comorbidity severity grades based on CCI score		
Mild		11 (10.5)
Moderate		40 (38.1)
Severe		54 (51.4)
Length of hospital stay (in days), Median (IQ)		10 (6, 15)
Patients who took medication in the past 03 months		71 (47.0)
In-hospital medications		
**ATC code**	**Medications category according to ATC**	
A	Alimentary tract and metabolism	85 (56.3)
B	Blood and blood-forming organs	92 (60.9)
C	Cardiovascular system	113 (74.8)
H	Systemic hormonal preparations	31 (20.5)
J	Anti-infective for systemic use	104 (68.9)
M	Musculoskeletal system	2 (1.3)
N	Nervous system	39 (25.8)
P	Antiparasitic products, insecticides, and repellents	1 (0.7)
R	Respiratory system	28 (18.5)
V	Various agents	3 (2.0)
In hospital medications		6 (4, 7)
Patients with polypharmacy (≥ 5 medications per patient)		38 (28.2)

*Abbreviations*: *ATC* Anatomical Therapeutic Chemical, *CCI* Charlson comorbidity index, *ICD-11* International Classification of Diseases 11th Revision, *IQ* Interquartile

### Inappropriate medication use and healthcare expenditures

Of the total, at least one PIM use was identified in 111 (73.5%) participants. Furosemide, 77 (43%), was the most frequently used PIM. Clinicians are recommended to strictly monitor sodium level during furosemide initiation and dose alteration in older adults. The weak opioid analgesic, tramadol, 25 (14.5%), was the second most frequently used PIM. In general, cautious use of tramadol is recommended in older patients due to its association with the risk of hyponatremia. On the other hand, tramadol use in older patients with CrCl < 30 ml/min is not recommended because of its CNS adverse effect, and it was used in one patient with severely compromised CrCl (Table 3).

**Table 3:** Prevalence of specific Beers’ PIMs with recommendations and rationale

Specific PIMs	Frequency (%)	Beers recommendation	Reason (s)
Furosemide	77 (43)	Use with caution	May exacerbate or cause SIADH or hyponatremia; monitor sodium level closely when starting or changing dosages in older adults
Tramadol	25 (14.5)	Avoid if CrCl < 30 = 1	CNS adverse effect
		Use with caution = 24	May exacerbate or cause SIADH or hyponatremia; monitor sodium level closely when starting or changing dosages in older adults
Spironolactone	22 (11.4)	Avoid in patients with CrCl < 30 = 2	Increased potassium
		Use with caution = 20	May exacerbate or cause SIADH or hyponatremia; monitor sodium level closely when starting or changing dosages in older adults
Ranitidine	17 (8.8)	Reduce dose if CrCl is < 50	Mental status changes
Cimetidine	13 (7.2)	Reduce dose if CrCl is < 50	Mental status changes
Metoclopramide	8 (4.7)	Avoid, unless for gastroparesis with a duration of use not to exceed 12 weeks except in rare cases	Can cause extrapyramidal effects, including tardive dyskinesia; the risk may be greater in frail older adults
Digoxin	4 (2.1)	Avoid this rate control agent as first-line therapy for atrial fibrillation	Should not be used as a first-line agent in atrial fibrillation, because there are safer and more effective alternatives for rate control
Aspirin and Warfarin	3 (1.5)	Avoid when possible; if used together, monitor INR closely	Increased risk of bleeding
Hydrochlorothiazide	3 (2.1)	Use with caution	May exacerbate or cause SIADH or hyponatremia; monitor sodium level closely when starting or changing dosages in older adults
Amitriptyline	2 (1.0)	Avoid	Highly anticholinergic, sedating, and cause orthostatic hypotension
Warfarin and Ciprofloxacin	2 (1.0)	Avoid when possible; if used together, monitor INR closely	Increased risk of bleeding
Aspirin	1 (0.5)	Use with caution in patients ≥ 70 years	Aspirin for primary prevention of cardiovascular disease
Dexamethasone and NSAID	1 (0.5)	Avoid; if not possible, provide gastrointestinal protection	Increased risk of peptic ulcer disease or gastrointestinal bleeding
Sliding-scale regular Insulin alone	1 (0.5)	Avoid	Insulin regimens that include only short-or rapid-acting insulin increase the risk of hypoglycemia without improvement in hyperglycemia management regardless of the care setting
Risperidone	1 (0.5)	Avoid	Avoid in older adults with or at high risk of delirium because of the potential of inducing or worsening delirium

*CrCl* Creatinine clearance, *NSAID* Nonsteroidal anti-inflammatory drug, *PIM* Potentially inappropriate medication, *SIADH* Syndrome of inappropriate secretion of antidiuretic hormone

**Fig. 1:**
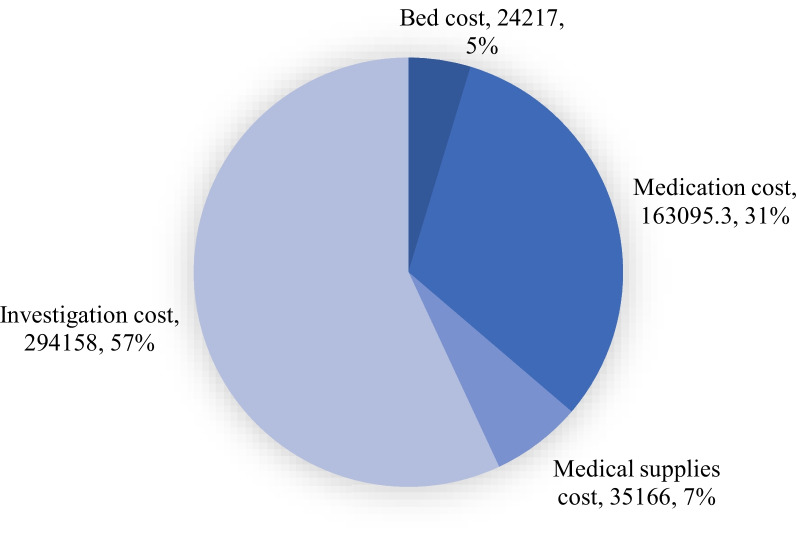
Healthcare expenditure of older adults in terms of expenditure category

Over their stay in the medical wards, the participants spent a total of 516,636.3 ETB for health care utilization (Fig. 1).

The expenditure was higher in those who took PIMs than in those who did not (385,368.6 ETB versus 131,267.7 ETB). The median expenditures for medical supplies (*p* = 0.025) and investigations (*p* = 0.033), as well as the total expenditure (*p* = 0.023), were significantly different across the number of PIMs (Table 4).

**Table 4:** PIMs and the distribution of healthcare expenditure

Number of PIMs		Healthcare expenditures, ETB				
	**Frequency (%)**	**Bed costs**	**Medication costs**	**Medical supply cost**	**Investigation costs**	**Total costs**
None	40 (26.5)	96 (72, 144)	824 (538.5, 1302.5)	210 (180, 280)	1564 (975, 1984)	2902.5 (2181.5, 3966.9)
One	59 (39.1)	120 (72, 156)	824 (442.9, 1265)	155 (110, 250)	1720 (1126, 2450)	2884 (2150, 3912)
Two	37 (24.5)	144 (84, 180)	824 (566, 1405)	233 (154, 328)	1800 (1281, 2350)	3051 (2410, 4055)
Three and above	15 (9.9)	166 (72, 240)	824 (630, 1680)	240 (130, 325)	2250 (1870, 2710)	4042 (3478.7, 4897)
***p*-value**		0.153	0.525	0.025*	0.033*	0.023*

^*^Statistically significant. Expenditures are in Ethiopian Birr (ETB). The exchange rate on 01 March 2023, 1$ = 57.7537 ETB

### Factors associated with older adult patients’ healthcare expenditure

In bivariate regression, a significant association was observed between the total healthcare expenditure of older adults and the number of PIMs prescribed (*p* = 0.044). However, this association was lost after adjusting for other factors in the multivariate regression analysis. A 30.7% and 19.6% decrease in healthcare expenditure was observed in patients with diseases of the nervous system (*p* = 0.002) and the respiratory system (*p* = 0.003), respectively, as compared to those without. On the other hand, each additional number of prescribed in-hospital medications (*p* < 0.001) and taking blood and blood-forming organ class of medication (*p* = 0.042) are associated with a 6.8% and 15.1% increase in healthcare expenditure (Table 5).

**Table 5:** Bivariate and multivariate generalized linear model regression analysis for identifying factors associated with the total healthcare expenditures of hospitalized older adults

Variables	Unadjusted B (95%CI)	*p*- value	Adjusted B (95%CI)	*p*-value
Number of PIMs (reference: No PIM)		**0.044**		
One PIM	-0.042 (-0.238, 0.153)	0.670	-0.162 (-0.329, 0.004)	0.056
Two PIMs	0.111 (-0.107, 0.328)	0.319	-0.174 (-0.369, 0.021)	0.080
Three and above	0.265 (-0.023, 0.554)	0.072	-0.028 (-0.283, 0.227)	0.832
Female sex (reference: Male)	-0.178 (-0.378, 0.022)	0.081	-0.181 (-0.265, -0.048)	0.175
Financial dependence (reference: Dependent)				
Independent	0.259 (0.076, 0.441)	**0.005**	-0108 (-0.257, 0.040)	0.153
Traditional medicine use (reference: no)	-0.176 (-0.401, 0.048)	0.124	-0.012 (-0.189, 0.166)	0.899
Patients with endocrine, nutritional or metabolic diseases (reference: without)	0.161 (-0.024, 0.345)	0.089	0.115 (-0.022, 0.251)	0.101
Patients with diseases of the nervous system (reference: without)	-0.307 (-0.533, -0.082)	0.008	-0.307 (-0.502, -0.112)	0.002*
Patients with diseases of the respiratory system (reference: without)	-0.126 (-0.282, 0.030)	0.114	-0.196 (-0.327, -0.065)	0.003*
Patients with symptoms, signs or clinical findings, not elsewhere classified (reference: without)	-0.183 (-0.474, 0.108)	0.217	-0.131 (-0.356, 0.094)	0.251
Number of in-hospital medications, count	0.087 (0.062, 0.113)	0.000	0.068 (0.035, 0.101)	< 0.001*
Patients with blood‒blood-forming organ medication prescription (reference: no)	0.304 (0.154, 0.453)	< 0.001	0.151 (0.005, 0.297)	0.042*
Patients with systemic hormonal medications prescription (reference: no)	0.146 (-0.049, 0.342)	0.142	-0.009 (-0.174, 0.156)	0.916
Patients with antiinfectives for systemic use prescription (reference: no)	0.247 (0.085, 0.409)	0.003	0.113 (-0.034, 0.260)	0.133
Patients with nervous system medications prescription (reference: no)	0.179 (0.0003, 0.357)	0.050	0.045 (-0.102, 0.191)	0.550
Length of hospital stay, days	0.007 (0.002, 0.012)	0.010	0.003 (-0.001, 0.007)	0.146

^*^Statistically significant

## Discussion

In the present study, relevant information was captured with the primary intention to examine the existence of an association between the healthcare expenditures of older adults and PIM use. Additionally, PIM prevalence, specific types of PIMs prescribed, specific categories of healthcare expenditures, and other factors associated with total healthcare expenditure were also determined as secondary outcomes. To the investigators` knowledge, this study is a pioneer in assessing the association between healthcare expenditure and the frequency of PIM use in Ethiopia.

In the present study, the overall healthcare expenditure of participants was 516,636.3 ETB. The unadjusted expenditure in PIM users exceeds the expenditure in non-PIM users by 254,100.9 ETB (385,368.6 ETB versus 131,267.7 ETB), implying that econometric studies in the healthcare system should also consider PIM use as part of the multiple factors that increase healthcare expenditures. Consistent findings were reported in various studies from different geographical regions [13, 15–17, 27, 41].

From the five categories of healthcare expenditures assessed in the present study, the median unadjusted expenditures for medical supplies (*p* = 0.025), investigations (*p* = 0.033), and total expenditure (*p* = 0.023) were significantly higher in those patients with two or more PIMs than in those with no or one PIM. Despite the difference in PIM assessment criteria, settings, periods, cost categories considered, and study designs, partly similar findings have been recorded in studies from the United States [13, 41, 42], Japan [16], Germany [17], and Finland [27]. They reported significantly higher medication/pharmacy [13, 16, 42], medical supplies [17], and total costs [13, 15–17, 27, 41] in PIM users than in non-PIM users. In the present study, the median length of hospital stay (10 days versus 8.5 days), medications prescribed (7 versus 5), and diseases diagnosed (4 versus 3) were higher in PIM users than in non-PIM users. These differences in the patterns of healthcare resource utilization can explain the higher healthcare expenditures seen in PIM users than in non-PIM users.

In some previous studies, PIM utilization is identified as a significant predictor of higher healthcare expenditure [13, 14, 41]. However, in the present study, the association between PIM use and healthcare expenditure was inconclusive after controlling for other covariates. The small sample size employed in this study and the difference in PIM assessment criteria, settings, periods, cost categories considered, and study designs might have contributed to the discrepancy. The healthcare expenditure was 30.7% and 19.6% lower in patients with diseases of the nervous system (*p* = 0.002) and respiratory system (*p* = 0.003) compared to those without, respectively. From medication information, a 15.1% and 6.8% increase in healthcare expenditure was noted in patients taking blood and blood-forming organ class of medication (*p* = 0.042) and for one additional prescribed in-hospital medication (*p* < 0.001), respectively. This implies that assessing the possibility of deprescribing medications may help reduce healthcare expenditures in older adults.

This study has several strengths and limitations. The prospective nature of the study, a novelty in the local area, the use of updated Beers criteria by time, and the inclusion of several relevant independent variables are among the strengths of the present study. The limited sample size employed and failure to consider other categories of expenditures, such as indirect costs, professional costs, and costs of program medications in the country, such as antiretroviral and anti-tuberculosis medications, could be mentioned as drawbacks of the present study.

## Conclusion

The total healthcare expenditure is nearly three times higher in PIM users than in non-PIM users. However, the frequency of PIM use failed to predict the total healthcare expenditures after model adjustment. A significant decrease in total healthcare expenditure is observed in patients with diseases of the nervous system and diseases of the respiratory system. On the other hand, taking blood and blood-forming organ class of medication, and additional prescriptions significantly increased the total healthcare expenditure. Deprescribing medications, including those from blood and blood-forming organ classes, after evaluating the benefit-risk ratio may help to reduce the healthcare expenditures of older adult patients. Furthermore, similar, adequately powered, comparative study is warranted to identify the actual effect of PIM use on healthcare expenditures in the local context.

## Data Availability

The datasets used and/or analysed during the current study are available from the corresponding author upon reasonable request.

## Abbreviations

ADL: Activity of daily living
ATC: Anatomical therapeutic chemical
ETB: Ethiopian birr
CCI: Charlson comorbidity index
CrCl: Creatinine clearance
GDS: Geriatric depression scale
ICD-11: International classification of diseases 11th revision
IQ: Interquartile
JUMC: Jimma University medical centre
